# Relationship between inclusion level of *Vachellia tortilis* leaf meal and behavioral activities of finishing pigs

**DOI:** 10.5713/ajas.18.0884

**Published:** 2019-02-14

**Authors:** Fortune Thabethe, Mbongeni Khanyile, Cyprial Ndumiso Ncobela, Michael Chimonyo

**Affiliations:** 1 Animal and Poultry Science, University of KwaZulu-Natal, P. Bag X01 Scottsville 3209, Pietermaritzburg, South Africa; 2 Department of Agriculture, Faculty of Science and Agriculture, University of Zululand, KwaDlangezwa 3886, South Africa

**Keywords:** Fiber, Proanthocyanidins, Relationship, Time Spent

## Abstract

**Objective:**

The study was conducted to establish a relationship between inclusion level of *Vachellia tortilis* (*V. tortilis*) leaf meal and time spent on different behavioral activities by finishing pigs.

**Methods:**

A total of forty-eight male Large White×Landrace finishing pigs with a mean (±standard deviation) body weight of 63.8±3.28 kg aged 14 wks were assigned to individual pens in a completely randomized design. Pigs were fed on diets containing 0, 30, 60, 90, 120, and 150 g/kg dry matter of *V. tortilis* leaf meal *ad libitum* with fresh water provided throughout the trial. There were eight pigs in each experimental diet. The behavior of pigs was observed for three wks twice a wk from 0600 to 1800 h using six closed circuit television cameras.

**Results:**

Increasing levels of *V. tortilis* leaf meal caused a linear decrease (p<0.05) in time spent eating, lying down and the number of visit to the feeder. Time spent standing and biting objects increased linearly (p<0.05) with increasing inclusion level of *V. tortilis* leaf meal. The was a negative linear relationship (p<0.05) between condensed tannins versus time spent eating, lying down and number of feeder visits. Condensed tannins showed a positive linear relationship (p<0.05) with time spent standing and biting objects. Neutral detergent fiber caused a linear decrease (p<0.05) in number of feeder visits, time spent eating, time spent standing.

**Conclusion:**

Inclusion level of *V. tortilis* leaf meal reduces time spent eating, lying down and the number of feeder visit while prolonging time spent standing and biting of objects. Condensed tannins and dietary fiber are among nutritional factors affecting behavioral activities displayed by finishing pigs.

## INTRODUCTION

In tropical regions, there is a growing trend towards the use of leaves from leguminous trees as protein source in pig diets [1]. This is propelled by climate change and poor vegetation management practices, which increases the dominance of shrub legumes compared to grasses in rangelands [2]. The use of leguminous leaves is also stimulated by escalating prices of protein-rich conventional feedstuffs such as soybean due to competition with human. Leguminous trees such as *Vachellia tortilis* (*V. tortilis*) are abundantly available, accessible and have reasonable nutrient profile [1,2]. *V. tortilis* has high levels of crude protein that ranges from 160 to 218 g/kg dry matter (DM) [3], which makes it appropriate to be used as a source of amino acids. It is also rich in neutral detergent fiber (NDF, 324 to 373 g/kg DM), acid detergent fiber (ADF, 138 to 171 g/kg DM) and polyphenols such as condensed tannins (2.61 to 12.9 mg/kg DM) [1]. Except growth performance [4], welfare aspect of using *V. tortilis* in finishing is often ignored yet it helps to broadly understand the worth of utilizing this feedstuff in pigs.

There is a compelling evidence that finishing pigs fed fibrous diet spend more time ingesting their daily diet and displaying stronger mastication movements [5,6]. The amount of condensed tannins (also known as proanthocyanidins) and fiber present in *Vachellia* leaf meals may also influence behavioral activities of finishing pigs [4,6]. V*. tortilis* contains proanthocynadins, which are plant-derived flavonoid polymers, bind with and engulf protein [7] thus compromising protein availability, digestibility and utilisation and this may have an indirect bearing on behavior of pigs [4]. To maximize the utilization of *V. tortilis* as feedstuff for pigs, information on the relationship between *V. tortilis* leaf meal against condensed tannins coupled with fiber is indispensable. Very few, if any, studies conducted to investigate the relationship between *V. tortilis* against behavioral activities. High inclusion of *V. tortilis* causes an astringent taste [1,8], thereby compromises growth performance, prolonging adaptation to the diet [4] and may influence feeding and stereotypic behavior in pigs. The current study helps in providing information about the homeostatic and welfare status of finishing pigs fed varying levels of *V. tortilis*. It also helps to gauge optimum inclusion levels of *V. tortilis* without undermining behavioral activities. It was hypothesized that *V. tortilis* leaf meal inclusion has no relationship with time spent on behavioral activities of pigs. The objective of the study was to determine the relationship between incremental levels of *V. tortilis* leaf meal and time spent on behavioral activities of finishing pigs.

## MATERIALS AND METHODS

### Description of study site and collection of *Vachellia tortilis* leaves

The study was conducted at Ukulinga Research Farm, University of KwaZulu-Natal (UKZN), Pietermaritzburg, South Africa. The farm is positioned 29°24′E and 30°24′S having an altitude of 775 m above sea level. The daytime mean temperatures ranges from 28°C to 43°C. The vegetation at the farm consists of several types of trees and grass species which include *Vachellia* species. The average rainfall is 735 mm. *V. tortilis* leaves were hand harvested at Makhathini Research Station, Jozini, South Africa. The leaves were harvested between April and May 2016 during post rainy season at an advanced stage of maturity according to the method described by Khanyile et al [1]. Briefly, leaves were harvested green, air-dried under shade by spreading on polyethylene sheets at room temperature for 72 h.

### Ethical consideration

The care and use of pigs were done according to the ethical guidelines stated by the certification of authorization to experiment on living animals given by the UKZN animal ethics committee: (Reference Number: AREC/101/015D).

### Pigs and housing management

A total of 48 clinically healthy male F1 hybrid (Large White× Landrace) pigs of 14 wks of age, were used in the study. The mean (±standard deviation) body weight of the pigs was 63.8 ±3.28 kg. The pigs were obtained from Kanhym farm in KwaZulu-Natal, South Africa, where they were housed in groups, to Ukulinga Research Farm (UKZN), where they were housed in individual pens. Prior to arrival, the pig house was disinfected with a natural disinfectant. A footbath was placed at the entrance of the pig house for biosecurity measures. The pigs were reared on individual pens having barren slatted floors measuring 2.1×1.1 m^2^. There was no any form of environmental enrichment in the pens and surroundings that would disturb behavior of pigs during the experiment. The pig barn had five rows of 16 pens. Of these, 3 rows of 16 pens were used to individually house pigs. Pigs were able to see each other across the row and next to each other. Prior to the diet treatment, pigs were all fed commercial diet without *V. tortilis*. Artificial lights were automatically switched on and off from 1800 h and 800 h, respectively. The house had raising curtains on both sides that were opened at 0845 h and closed at 1630 h. All pigs had free access to clean fresh water which was provided through low pressure nipple drinkers. They also had *ad libitum* access to feed which was offered through pre-weighed plastic self-feeder troughs (Big Dutchman Lean Machine, Postfach, Vechta, Germany).

### Experimental design and diets

Eight pigs were allocated using a completely randomized design into each of the six experimental diets. Each pig was used an experimental unit. Pigs had already adapted to the diet. The adaptation consisted of 10 d of adaptation experiment and 21 d of growth performance experiment [4]. The inclusion levels tested were 0, 30, 60, 90, 120, and 150 g/kg DM of *V. tortilis* leaf meal. Maximum inclusion level of leaf meal was determined by the digestibility estimates of energy and amino acids [1]. The diets were formulated to be isoproteinic and isocaloric using Winfeed (Winfeed Limited, Cambridge, UK) feed formulation software. Vitamins and mineral were supplemented to meet NRC recommended specification for finishing pigs [9]. Pigs were not supplemented with growth promoters. Table 1 is showing the ingredient composition of experimental diets. Average daily feed intakes of finishing pigs fed on 0, 30, 60, 90, 120, 150 g/kg DM of inclusion level of *V. tortilis* were 2.97, 2.96, 3.08, 2.82, 2.73 and 2.76 kg/d, respectively [4]. The average daily gain was 1.01, 101, 103, 0.96, 0.91, and 1.02 kg/d, respectively.

### Chemical composition of diets

Before analyzing the experimental diets, samples from each diet were ground through a 2 mm sieve at Ukulinga Research Farm, Pietermaritzburg. After milling, the samples were analyzed in triplicate, at the Animal and Poultry Science Laboratory at UKZN, Pietermaritzburg. Briefly, DM content was determined by the oven drying method, samples were dried for 4 d at 65°C. Dry samples were incinerated at 550°C overnight for ash content determination according to method 990.05 [10]. The dried samples were subjected to bomb calorimetry to determine gross energy. Ether extract was determined using the Soxhlet apparatus following method 920.39 described by AOAC [10]. Crude protein content was calculated using the formula: N×6.25, were nitrogen content was determined following the Dumas Combustion method in a Leco Truspec Nitrogen Analyser, St. Joseph, MI, USA by method 990.3 of AOAC [10]. The NDF and ADF were determined using the Ankom Fiber Analyzer (Ankom Macedon, NY, USA) according to [11]. The NDF was analysed using heat stable α-amylase (sigma A3306; Sigma Chemical Co., St. Louis, MO, USA).

The water holding capacity (WHC) was determined following methods described by Whittemore et al [12]. Briefly, 0.5 g of each feed sample was placed into a 50 mL centrifuge machine and distilled water of 25 mL was added. The tubes were wrapped and shake for 24 h and centrifuged at 6,000×g for 15 min at 20°C. The formed supernatant was discarded and the new weight of the sample was recorded. After freeze-drying, the weights of the recollected fluids were calculated from the difference between the new sample and the dried sample. The weight of the recollected fluid was divided by the weight of the dried sample to determine the WHC of the feed sample, which was then expressed in g water/g of dry material. Bulk density was determined using the water displacement method as described by Kyriazakis and Emmans [13]. For mineral analyses, ground samples were ashed at 550°C overnight and dissolved in a 1 M HCL [14], then analysed using the Varian 720 Inductively Coupled Plasma Emision Spectrometer (ICP- OES, Frankfurt, Germany) with an atomic absorption. Proanthocyanidins content was detected calorimetrically by the butanol-HCL process described by Reed et al [15]. For amino acids, acid hydrolysis was used according to method 982.30 described by AOAC [10]. Before analyses via amino acid analyser (SY-KAM, Erising, Gewerbering, Germany), modifications were made as explained by Mills et al [16]. The chemical composition of the diets is given in Table 2.

### Measuring behavioral activities

Behavioral activities were observed on individual pigs for three wks, twice a wk from 0800 am to 1800 h, using six motorized indoor closed circuit television cameras. Cameras were installed in different locations in the housing pen such that each camera appropriately captured eight pigs. All cameras were attached to a digital video recorder control system and transcend one terabyte external hard drive. The use of video cameras was adopted to avoid disturbances during behavioral data collection. Behavioral activities recorded were time spent eating, drinking, standing, lying down, sniffing, biting, licking objects and the number of visits to the feeder. The description of behavioral activities is given in Table 3. As described by Bakare et al [6], time spent on each behavioral activity was divided by body weight of a pig. This was done to account for variation that could be caused by body weight when measuring behavioral activities.

### Statistical analyses

The PROC UNIVARIATE procedure of SAS [17] was used to determine the normality of the data for time spent on each behavioral activity (eating, feeder visit, drinking, lying down, standing, sniffing, object biting, and licking). Then data for time spent on each behavioral activity were normalized using logarithmic transformation since it was not normally distributed. The PROC CORR procedure of SAS [17] was used to determine the correlation between each behavioral activity and the chemical components *V. tortilis* leaf meal inclusion. The PROC RSREG procedure of SAS [17] was used to determine relationship between inclusion levels of *V. tortilis* leaf meal against time spent on each behavioral activity. The regression analysis was also used to relate behavioral activities and chemical components of *V. tortilis* leaf meal.

$$\text{The regression model used was: Y}=\beta_{0}+\beta_{1}\text{V}+\beta_{2}{\text{V}}^{2}+\text{E}$$

Where: Y is the response variables (time spent eating, drinking, lying down, standing, sniffing, object biting and licking and the number of feeder visits); β_0_, β_1_, β_2_ regression coefficients; V is the inclusion level of *V. tortilis* leaf meal; E is the residual error.

## RESULTS

### Correlation coefficients between behavioral activities of pigs and chemical components of the feeds

The correlation coefficients among each behavioral activity are shown in Table 4. Time spent eating was positively correlated (p<0.05) to the number of visits to the feeders. A negative correlation (p<0.05) was observed between the number of feeder visits and time spent standing and sniffing walls in the pens. Time spent standing was positively correlated (p<0.05) with time spent sniffing the walls, biting and licking of objects. Time spent lying down was not correlated to any behavioral pattern. Time spent sniffing the walls was positively correlated (p<0.05) to time spent biting objects. A positive correlation (p<0.05) was observed between time spent biting and licking objects. The number of feeder visits were positively correlated (p<0.05) with time spent standing, sniffing the walls and biting objects. Table 5 shows the correlation coefficients of condensed tannins and fiber of *V. tortilis* leaf meal against behavioral activities. There was a negative correlation (p<0.05) between ADF content and time spent standing, sniffing walls and biting objects. The NDF showed a negative correlation (p<0.05) with time spent eating. Condensed tannins were positively correlated (p<0.001) with time spent sniffing walls, biting and licking objects and negatively correlated with time spent eating, drinking, number of feeder visits (p<0.001), standing and lying down (p<0.05).

### Relationship between increasing level of *Vachellia tortilis* leaf meal and time spent on behavioral activities of pigs

**Figure 1:**
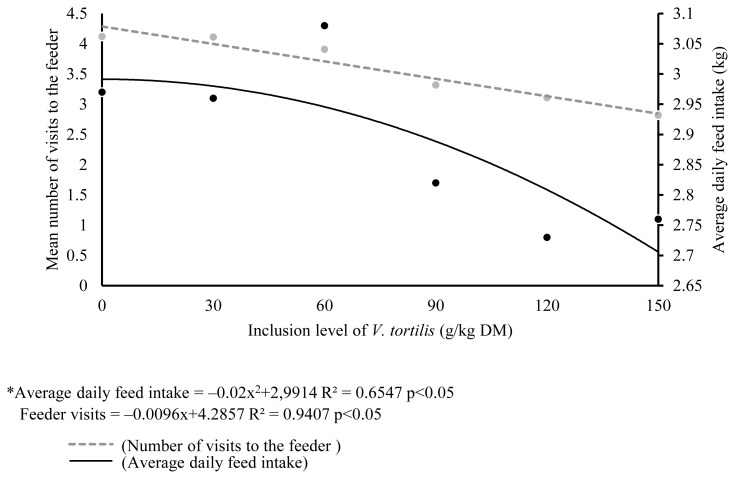
Response in number of visits and average daily gain against inclusion levels of *Vachellia tortilis*. * Values of average daily feed were adapted from Thabethe et al [4].

Table 6 shows the relationship on time spent on behavioral activities with increasing levels of *V. tortilis* leaf meal. A linear decrease (p<0.05) in time spent time eating and lying down was observed with increasing level of *V. tortilis* leaf meal. The time spent standing and biting objects increased linearly with increasing level of *V. tortilis* leaf meal (p<0.05). There was no relationship in time spent on drinking, sniffing walls and licking of objects with increasing level of *V. tortilis* leaf meal. The regression equations of behavioral activities are shown in Table 7. Number of visits to the feeder decreased linearly (p<0.0.5) with increasing levels of *V. tortilis* (Figure 1). There was a quadratic response in average daily feed intake versus inclusion level of *V. tortilis*. The relationship between *V. tortilis* leaf meal against condensed tannins and fiber is shown in Table 8. The negative linear relationship (p<0.05) between condensed tannins versus time spent eating, number of feeder visits, lying down was observed. Condensed tannins also exhibited a positive linear relationship (p<0.05) with time spent standing and biting objects. The NDF caused a linear decrease (p<0.05) in number of feeder visits, time spent eating, time spent standing. In the meantime, NDF linearly increased (p< 0.05) time spent biting objects. Time spent standing, lying down and biting objects decreased in a negative linear fashion (p<0.05) in response to ADF.

## DISCUSSION

To understand welfare aspect and feeding value of *V. tortilis* leaf meal in pigs, it is pertinent to not only conclude by determining feed intake and growth performance [1,4] but also changes in behavioral activities are crucial. To the best of our knowledge, behavioral response of pigs to tanning-containing feedstuff are reported for the first time. Finishing uncastrated male pigs (boars) are important for swine breeding programs and production. Photoperiod has a direct impact on behavioural activities in pigs [18]. Therefore, behavior was not recorded from when artificial light was used (1800 h to 0800 h). In addition, majority of pigs in the study were inactive after 1800 h and started to be active around 0800 h the next morning when personnel were arriving to provide new feed [6] Hence, recordings during inactive time were not done. A positive correlation (both feeder visits and time spent decreased) between time spent eating and number of visits could be due to inherent property of tannins from *V. tortilis*. Condensed tannins diminish weight gain and reduce feed efficiency by inhibiting protein digestion and releasing astringent taste [2,19]. A positive correlation between number of feeder visits and time spent drinking was anticipated. Apart from addressing hydration requirement, drinking is responsive to feeding. A negative correlation between number of visits to the feeder against time spent standing, sniffing walls and licking objects suggest that finishing pigs were exploring non-stereotypic behaviors such as drinking after visiting the feeder. A lack of correlation between time spent eating against other behavioral activities was unanticipated. This may mean that inclusion level of *V. tortilis* resulted to unmeasured behaviors such as time spent mounting walls, rubbing against the walls, sitting and walking around in the pen. These behavioral activities may symbolize uncomfortability of pigs due to reduction in appetite as a result of *V. tortilis*.

There was a negative correlation between ADF against time spent standing, sniffing walls and biting objects. The ADF is ingestible component of the feed, which consists of cellulose and lignin. A temporal fullness of the gut due to distension can be accountable for reduced time spent on behavioral activities such as sniffing walls and biting objects [20]. The NDF contains hemicellulose, lignin and cellulose, of which hemicellulose is partially fermented by gastrointestinal microbiota that produces exogenous enzymes in the gut of pigs [6]. The observed negative correlation between NDF versus time spent eating and number visits to the feeder could be related to hydrophilic polysaccharides, such as hemicellulose, found in *V. tortilis* that can absorb water and hold it in the lumen of the gut [21]. De Leeuw et al [20] reported high absorption, swelling, stomach wall distension in pigs consuming feed with high WHC, which results to satiety and henceforth number of visits to the feeder and time spent eating are reduced. A positive correlation between NDF and time spent standing, sniffing walls and biting objects was mainly due to a decrease in feeder visits and time spent eating.

Proanthocyanidins showed a negative correlation with time spent eating and feeder visits, suggesting that finishing pigs struggled to withstand the deleterious effects of the condensed tannins found in *V. tortilis*. Diets that are rich in condensed tannins give rise to astringent sensation in the mouth, thereby inducing negative feedback, prompting pigs to reduce feed consumption [22]. This sensation also reduces feed palatability and nutrient digestibility [19]. A positive correlation between condensed tannins and time spent standing, sniffing walls, biting and licking objects is entirely explained by a compromise time spent eating and visit to the feeder, which subject pigs into stereotypic behaviors. In contrast, Day et al [23] indicated that decreased in stereotypic behavioral activities is among positive effect of caused by condensed tannins. This largely depends upon the type of leguminous tree used, content of tannins available and its molecular weight.

Surprisingly, a negative linear relationship between time spent eating and levels of *V. tortilis* was observed. As reported on growth performance study [4], a quadratic decrease instead of linear was expected, where pigs would take some time to consume feed containing tannin-rich *V. tortilis* until a certain inclusion level where optimum time spent eating and feeder visits are reduced. A linear decrease suggests that *V. tortilis* inclusion drastically gave rise to astringent taste, a dryness flavor coupled with sour or bitter flavor that is normally produced by condensed tannins in the leaves. This irritable taste deterred pigs from eating feed containing *V. tortilis*, thus reducing the duration eating. Astringent taste is known to reduce feed intake and palatability [1,24].

A suppression of appetite and promotion of feed refusal caused by condensed tannins [2] may be the reason of linear decrease in number feeder visits with increasing levels of *V. tortilis*. In a growth performance study conducted by Thabethe et al [4] on finishing pigs, average daily feed intake showed a quadratic relationship with increasing levels of *V. tortilis*. A linear decrease in number of feeder visits was, therefore difficult explain. It could be due to condensed tannins that subjected finishing pigs to consume more feed containing *V. tortilis* (by virtue of large body capacity) in each visit to the feeder to meet their requirements until the feed intake is supressed. Condensed tannins are widely recognized as anti-nutritional factors to animals [19]. Pigs have a limited ability to utilise tannin-rich feedstuff due to their physiological nature. It is because, unlike ruminants, they do not have bacteria, which counter the effects of tannins [2]. This also explains a linear decrease in feeder visits against condensed tannins. A negative linear relationship between time spent eating and feeder visits against NDF was tricky to explain. This is because it is accepted that an increase in fibrous property (NDF) of *V. tortilis* reduces nutrient density, thus promoting feed intake and time spent eating to meet their nutrient requirement until the gut capacity is reached, then feed intake and time spent eating is reduced [6]. The linear decrease in time spent eating against NDF could be due to fact that finishing pigs have a facility to efficiently utilize fiber by virtue of hindgut fermentation.

A linear decrease in time spent lying down with increasing levels of *V. tortilis* may be chiefly related to a decrease in time spent eating and visiting the feeder. Lying down is often the reaction after feeding behavioral activities such as time spent eating and drinking. A decrease in time spent lying down could, therefore, be due to frustration as result of decrease time spent eating and feeder visits. A linear increase in time spent standing and biting objects might be attributed to linear decrease in time spent lying down. Substrate-directed behaviors occur when there is a poor palatability of the feed. However, palatability was not measured in the current study. Linear behavioral responses of finishing pigs found in the present study preclude from estimating optimal inclusion levels of *V. tortilis*.

## CONCLUSION

Inclusion level of *V. tortilis* leaf meal diets alters the behavioral activities of finishing pigs. It reduces time spent eating, lying down and the number of feeder visit while prolonging time spent standing and biting of objects. It is understood that changes in behavioral activities are due to condensed tannins, NDF and ADF. To maximize utilization of *V. tortilis*, determination of the behavioral activities of finishing pigs fed on *V. tortilis* leaf meal treated with polyphenolic-binding agents is essential.

## Figures and Tables

**Table 1:** Ingredient composition of experimental diets (g/kg DM)

Ingredient (g/kg)	Vachellia tortilis inclusion level (g/kg DM)					

	0	30	60	90	120	150
Maize	458	432	406	382	357	333
Wheat bran	356	346	337	326	315	304
Soybean 46	86	91.2	94.4	101	106	111
*Vachellia tortilis* leaves	0	30	60	90	120	150
Oil-sunflower	60.1	61.1	62.0	62.9	63.8	64.8
Limestone	20.9	20.4	19.9	19.5	18.9	18.2
Monocalcium phosphate	9.61	9.9	10.2	10.5	10.8	11.1
Salt	4.9	5.0	5.02	5.03	5.03	5.05
Vitamin-mineral premix1)	1.5	1.5	1.5	1.5	1.5	1.5
L-lysine-HCL	1.64	1.44	1.24	1.05	0.85	0.65
Threonine	0.97	0.86	0.75	0.64	0.52	0.4
Methionine	0.38	0.42	0.46	0.5	0.54	0.58

DM, dry matter.

1) Provides (/kg of DM of diet): vitamin A, 4.8 mg; vitamin D_3_, 0.09 mg; vitamin E, 50 mg; vitamin K_3_ (43%), 1.0 mg; vitamin B_1_, 1.6 mg; vitamin B_2_, 2.6 mg; niacin (99.5%), 33.6 mg; vitamin B_12_, 0.01 mg; vitamin B_6_ 98%, 2.0 mg; choline (chloride 60%), 121 mg; folic acid (96% pure), 0.48 mg; biotin, 0.18 mg; calcium pantothenate (98%), 5.2 mg; zinc balitracin, 90.0 mg; manganese sulphate, 120.0 mg; zinc, 100 mg; copper, 8 mg; potassium iodide (Iodine 76.45%), 0.4 mg; cobalt sulphate, 0.2 mg; ferrous sulphate, 100.0 mg; and selenium, 0.32 mg on dolomite carrier.

**Table 2:** Nutrient composition of experimental diets

Component	*Vachellia tortilis* inclusion level (g/kg DM)					

	0	30	60	90	120	150
DM (g/kg)	946	935	899	940	897	896
GE (MJ/kg)	17.6	17.5	17.3	17.3	17.4	17.2
Ash (g/kg DM)	90	90	90	89	89	86
CP(g/kg DM)	144	140	142	146	146	148
EE(g/kg DM)	114	104	109	114	113	116
Starch (g/kg DM)	318	321	305	274	239	225
ADF (g/kg DM)	137	136	139	145	145	147
NDF (g/kg DM)	303	313	321	339	348	356
Lysine (g/100g DM)	10.3	10.1	10.1	10.5	11.0	10.7
Threonine (g/100g DM)	7.2	7.2	7.5	8.0	7.5	8.0
Methionine (g/100g DM)	5.0	4.3	4.5	4.3	4.8	5.0
CT (mg/kg DM)	-	2.3	3.1	5.1	6.6	7.9
BD (mL/g DM)	1.31	1.52	1.59	1.63	1.64	1.69
WHC (g_water_/g_feed_ DM)	2.27	2.4	2.61	2.88	3.23	3.44
Calcium (g/kg DM)	10.1	15.4	16.8	17.3	17.2	19.8
Phosphorus (g/kg DM)	7.3	9.7	10.1	10.7	10.3	11.9
Magnesium (g/kg)	9.8	8.9	10.1	10.1	9.3	10.1
Potassium (g/kg)	8.8	9.0	9.3	10.2	10.4	11.1
Sodium (g/kg)	2.3	5.3	10.2	10.1	9.8	12.3
Zinc (mg/kg)	90.8	91.1	88.3	81.2	82.3	78.2
Copper (mg/kg)	8.9	7.8	9.14	8.15	7.23	7.31
Manganese (mg/kg)	124	111	109	124	123	128
Iron (mg/kg)	146	201	304	338	344	359

DM, dry matter; GE, gross energy; CP, crude protein; EE, ether extract; ADF, acid detergent fiber; NDF, neutral detergent fiber; CT, condensed tannins; BD, bulk density; WHC, water holding capacity.

**Table 3:** Ethogram of recorded behavioral activities

Behavior	Description of behavior
Eating	Feed consumption from the feeder / snout in contact with the feeder
Drinking	Manipulating the nipple / snout in contact with the nipple drinker
Feeder visit	When a pig goes to the feeder
Standing	Body supported by four legs without stamping
Lying down	Lying ventral or lateral with sternum in contact with the floor
Sniffing	When sniffing is performed against the pen floor
Biting	When biting is performed against the pen objects (chain and sides of the pen)
Licking	When licking is performed against the pen objects

**Table 4:** Pearson’s correlation coefficients among behavioral activities of finishing pigs fed *Vachellia tortilis* leaf meal

Items	ET	DK	FV	ST	LY	SN	OB	OL
ET	-	1.18^NS^	0.24*	0.08^NS^	−0.02^NS^	0.09^NS^	−0.04^NS^	−0.00^NS^
DK	-	-	0.37**	0.03^NS^	0.84^NS^	0.32^NS^	0.27^NS^	0.10^NS^
FV	-	-	-	−0.21*	0.09^NS^	−0.23*	0.11^NS^	−0.01^NS^
ST	-	-	-	-	−0.07^NS^	0.47**	0.62**	0.43**
LY	-	-	-	-	-	0.02^NS^	0.01^NS^	−0.12^NS^
SN	-	-	-	-	-	-	0.29*	0.01^NS^
OB	-	-	-	-	-	-	-	0.36*
OL	-	-	-	-	-	-	-	-

ET, time spent eating; DK, time spent drinking; FV, number of feeder visit; ST, time spent standing; LY, time spent lying down; SN, time spent sniffing; OB, time spent biting objects; OL, time spent licking objects.

Significance level:

*** p<0.001;

** p<0.01;

* p<0.05;

NS, not significant (p>0.05).

**Table 5:** Pearson’s correlation coefficients of behavioral activities of finishing pigs against condensed tannins and fiber of Vachellia tortilis leaf meal

Components	ET	DK	FV	ST	LY	SN	OB	OL
Acid detergent fiber (g/kg DM)	0.18^NS^	−0.16^NS^	0.09^NS^	−0.27*	0.16^NS^	−0.28*	−0.30*	0.33^NS^
Neutral detergent fiber (g/kg DM)	−0.29*	0.19^NS^	−0.21*	0.26*	−0.13^NS^	0.28*	0.35*	0.35^NS^
Condensed tannins (mg/kg DM)	−0.50**	−0.44	−0.47**	0.37*	−0.45^NS^	0.49**	0.38*	0.42*

DM, dry matter; ET, time spent eating; DK, time spent drinking; FV, number of feeder visit; ST, time spent standing; LY, time spent lying down; SN, time spent sniffing; OB, time spent biting objects; OL, time spent licking objects.

Significance level

*** p<0.001;

** p<0.01;

* p<0.05;

NS not significant (p>0.05).

**Table 6:** Relationship between time spent on behavioral activities with increasing level of Vachellia tortilis leaf meal

Activity (min/d/BW)	Vachellia tortilis inclusion level (g/kg DM)						SEM	Polynomial regression	
	
	0	30	60	90	120	150		Linear	Quadratic
ET	2.31	2.22	2.21	2.21	2.21	2.17	0.01	*	NS
DK	1.64	1.58	1.53	1.67	1.66	1.58	0.02	NS	NS
ST	1.31	1.25	1.37	1.38	1.71	1.39	0.01	*	NS
LY	2.47	2.47	2.41	2.37	2.34	2.44	0.03	*	NS
SN	1.42	1.52	1.48	1.52	1.62	1.57	0.06	NS	NS
OB	0.08	0.74	0.92	0.87	1.31	1.04	0.12	*	NS
OL	0.97	0.75	0.92	0.87	1.31	1.04	0.11	NS	NS

BW, body weight; DM, dry matter; SEM, standard error of means; ET, time spent eating; DK, time spent drinking; ST, time spent standing; LY, time spent lying down; SN, time spent sniffing; OB, time spent biting objects; OL, time spent licking objects.

Significance level:

*** p<0.001;

** p<0.01;

* p<0.05;

NS, not significant (p>0.05).

**Table 7:** Regression equations of behavioral activities of pigs fed *Vachellia tortilis* leaf meal

Activity1)	R^2^	Equation	Significance levels
ET	0.16	ET = −0.051 (0.032)x–2.349 (0.048)	***
ST	0.07	ST = 0.107 (0.103)x+1.152 (0.156)	*
LY	0.05	LY = −0.101 (0.058)x+2.58 (0.089)	*
OB	0.13	OB = 0.0822 (0.113)x+0.67(0.172)	*

ET, time spent eating; ST, time spent standing; LY, time spent lying down; OB, time spent biting object.

Values in parentheses are standard errors of the estimates.

1) Behavioral activities displayed are only those showed statistical differences.

Significance level:

*** p<0.001;

** p<0.01;

* p<0.05;

NS not significant (p>0.05).

**Table 8:** Relationship between behavioral activities against condensed tannins and fiber on behavioral activities

Activity1)	Component	Regression	Significance level
ET	CT	Linear decrease	***
FV	CT	Linear decrease	*
ST	CT	Linear increase	*
LY	CT	Linear decrease	*
OB	CT	Linear increase	*
ET	NDF	Linear decrease	*
FV	NDF	Linear decrease	*
ST	NDF	Linear decrease	**
OB	NDF	Linear increase	**
ST	ADF	Linear decrease	**
LY	ADF	Linear decrease	*
OB	ADF	Linear decrease	*

ET, time spent eating; CT, condensed tannins; FV, number of feeder visit; ST, time spent standing; LY, time spent lying down; OB, time spent biting object; NDF, neutral detergent fiber; ADF, acid detergent fiber.

1) Behavioural activities displayed are only those showed statistical difference.

Significance level:

*** p<0.001;

** p<0.01;

* p<0.05;

NS, not significant (p>0.05).
